# Supplementary material for: The tumor suppressor PTPRK promotes ZNRF3 internalization and is required for Wnt inhibition in the Spemann organizer
Source: eLife. 2020 Jan 14;9:e51248. doi: 10.7554/eLife.51248 (PMC6996932; doi:10.7554/eLife.51248)
Supplement: Supplementary file 1. [file elife-51248-supp1.docx]

Morpholino antisense oligonucleotides (Mo, Gene Tools)

| **Name** | **Sequence (5’→3‘)** | **Source** |
| --- | --- | --- |
| Control Mo | CCTCTTACCTCAGTTACAATTTATA | Gene Tools |
| *ptprk* Mo | TTCTTACCTGCACACTTGGTTCTTG | This paper |
| *znrf3* Mo1 | CCACTTACCTGCACGATCTCCCCCT | This paper |
| *znrf3* Mo2 | AACATAATTTCCCAGTCCTCAGTGG | This paper |
| *lrp6* Mo | CCCCGGCTTCTCCGCTCCGACCCCT | Hassler et al., 2007 |
| *β-catenin* Mo | TTTCAACCGTTTCCAAAGAACCAGG | Heasman et al., 2000 |

Pimers (Sigma) used for cloning

| **Gene** | **Sequence (5’→3‘)** |
| --- | --- |
| *X. tropicalis* ptprk | Forward: CCCCCCGGGGAGCCTCCAAGGCCTATTGC  Reverse: CCCGAATTCGGATGGTAGTCCCTGGATGC |
| *X. tropicalis* znrf3 | Forward: ATAAGAATGCGGCCGCATGCACCCACTTGGACTCTGTAAT  Reverse: ACGCGTCGACGTCCTGAAGATGCATGGTCCAGT |
| *Human* pCS2-V5-PTPRK | Forward: CCC**TCCGGA**CAAGGCCAGTTCTCCGCAGGT  Reverse: CCC**CTCGAG**TTAAGATGATTCCAGGTACTCCAAAGC |
| *Human* pCS2-V5-PTPRKΔC | Forward: CCC**ATCGAT**GCCACCATGGATACGACTGCGGCGGC  Reverse: CCC**TCCGGA**TTTTACAATTAATATGACAACTAGGAG |
| *Human* pCS2-V5-PTPRK C1089S | 1^st^ PCR  Forward(1): AATAAGATCTTCTTGAACTGG  Reverse(1): CAGCACCAGCACTGGAATGTACAA  Forward(2): TTGTACATTCCAGTGCTGGTGCTG  Reverse(2): TACGCCGAGATCTTAAGGC  2^nd^ PCR  Forward(1): AATA**AGATCT**TCTTGAACTGG  Reverse(2): TACGCCG**AGATCT**TAAGGC |
| *Human* pCS2-V5-PTPRK D1057A | 1^st^ PCR  Forward(1): TTGGCTGTACAGGGATGGCT  Reverse(1): ATGGGCAGGCCAGCCCGTGAAATG  Forward(2): CATTTCACGGGCTGGCCTGCCCAT  Reverse(2): GCACTGCAATGTACAACGATG  2^nd^ PCR  Forward(1): TTGGC**TGTACA**GGGATGGCT  Reverse(2): GCACTGCAA**TGTACA**ACGATG |
| *Human*  *pcDNA4/TO-ZNRF3Δ4Y-HA* | Forward: Phos - TTCCAGGGCCTCAGCTACCCG  Reverse: Phos - CTGGGAGAAGCAAGCTGCCTT |
| *Human*  *pcDNA4/TO-ZNRF3(4YF)-HA* | Forward: Phos - CACTTCTTCTTCCAGGGCCTC  Reverse: Phos - CTGGAACATAGTTTCGAATTGTG |
| *Human* pCS2-V5-Frizzled5 | Forward: CCC**TCCGGA**GCGTCCAAGGCCCCGGTGTG  Reverse: CCC**TCTAGA**TTACACGTGCGACAGGGACACC |

Primers (Sigma) for synthesis of linear DNA template of sgRNA

| **Gene** | **Sequence (5’→3‘)** |
| --- | --- |
| Control sgRNA | Forward: GCAGCTAATACGACTCACTATAGGTTTTAGAGCTAGAAATA  Reverse: AAAAGCACCGACTCGGTGCCACTTTTTCAAGTTGATAACGGACTAGCCTTATTTTAACTTGCTATTTCTAGCTCTAAAAC |
| *X. tropicalis ptprk* sgRNA | Forward: GCAGCTAATACGACTCACTATAGTGTGGTGGTGCAATAGGCCTGTTTTAGAGCTAGAAATA (sgRNA sequence is wavily underlined)  Reverse: The same with contrl gRNA |

Primer for CRISPR genotyping

| **Gene** | **Sequence (5’→3‘)** |
| --- | --- |
| X.tropicalis *ptprk* | Forward: AGCCTCAGTCTGGCTTTTTAATTT  Reverse: CTCAAGGTTAACGCTACGAAAAATC |

Quantitative RT-PCR primers (Sigma) and UPL probe numbers (Roche)

| **Gene** | **Sequence (5’→3‘)** | **UPL probe** |
| --- | --- | --- |
| Human *AXIN2* | Forward: CCACACCCTTCTCCAATCC  Reverse: TGCCAGTTTCTTTGGCTCTT | 32 |
| Human *GAPDH* | Forward: GCATCCTGGGCTACACTGAG  Reverse: AGGTGGAGGAGTGGGTGTC | 82 |
| Human *LRP6* | Forward: GGCACTTACTTCCCTGCAAT  Reverse: TGTAATGTGATCGCTCTGTGG | 22 |
| Human *PTPRK* | Forward: TGAGGAACAGCAAGCCAAA  Reverse: TCCAAAGCTACATCATAGCAGAA | 31 |
| Human *RNF43* | Forward: GTTTGCTGGTGTTGCTGAAA  Reverse: TGGCATTGCACAGGTACAG | 67 |
| Human *ZNRF3* | Forward: TGTGCCATCTGTCTGGAGAA  Reverse: TTCCTGTGAAACCGGTGAGT | 17 |
| *X. tropicalis bf1* | Forward: GCAAGGGCAACTACTGGATG  Reverse: TGGTCCCACCAATGAACAC | 3 |
| *X. tropicalis lrp6* | Forward: TCCTCCAGCACTAAAGGAACC  Reverse: TCCATTGTGTAATGGGAACG | 10 |
| *X. tropicalis odc* | Forward: tttggtgccacccttaaaac  Reverse: ccactgccaacatggaaac | 50 |
| *X. tropicalis otx2* | Forward: tacatccgtcggtgggata  Reverse: gctctggtgaaagtggtcct | 82 |
| *X. tropicalis ptprk* | Forward: TTGGATCCAGACACGGAGTA  Reverse: GGGTTTTAGGTGTTCTCATAGGC | 2 |
| *X. tropicalis sox3* | Forward: agccatcacctcccacac  Reverse: caggtacatgctgatcatatctcg | 14 |
| *X. tropicalis znrf3* | Forward: CACACTACTGAGTTACAAGGGAGGT  Reverse: TCATCGTTGTTATTACAGAGTCCAA | 81 |

siRNA information (Horizon Discovery)

| **Gene** | **Cat#** |
| --- | --- |
| siGENOME SMARTpool human PTPRK siRNA | M-004204-01-0005 |
| siGENOME human PTPRK siRNA #2 | D-004204-02-0005 |
| siGENOME human PTPRK siRNA #5 | D-004204-05-0005 |
| siGENOME Non-targeting siRNA #1 | D-001210-01-20 |
| siGENOME SMARTpool human ZNRF3 siRNA | M-010747-02-0005 |
| siGENOME SMARTpool human RNF43 siRNA | M-007004-02-0005 |
| siGENOME SMARTpool human PTPRG siRNA | M-008069-00-0005 |
| siGENOME SMARTpool human PTPRA siRNA | M-004519-01-0005 |
| siGENOME SMARTpool human PTPRH siRNA | M-009448-01-0005 |
| siGENOME SMARTpool human PTPRF siRNA | M-008375-02-0005 |
| siGENOME SMARTpool human PTPRS siRNA | M-009662-02-0005 |
| siGENOME SMARTpool human PTPRJ siRNA | M-008476-02-0005 |
| siGENOME SMARTpool human PTPRM siRNA | M-006326-01-0005 |
| siGENOME SMARTpool human LRP6 siRNA | M-003845-03-0005 |
| siGENOME SMARTpool human β-catenin siRNA | M-003482-00-0005 |
